# iTTCA-RF: a random forest predictor for tumor T cell antigens

**DOI:** 10.1186/s12967-021-03084-x

**Published:** 2021-10-27

**Authors:** Shihu Jiao, Quan Zou, Huannan Guo, Lei Shi

**Affiliations:** 1 Yangtze Delta Region Institute (Quzhou), University of Electronic Science and Technology of China, Quzhou, China; 2 Institute of Fundamental and Frontier Sciences, University of Electronic Science and Technology of China, Chengdu, China; 3 Department of Oncology, General Hospital of Heilongjiang Province Land Reclamation Bureau, Harbin, China; 4 Department of Spine Surgery, Changzheng Hospital, Naval Medical University, Shanghai, China

**Keywords:** Tumor T cell antigens, Random forest, MRMD, Feature selection, Hybrid features

## Abstract

**Background:**

Cancer is one of the most serious diseases threatening human health. Cancer immunotherapy represents the most promising treatment strategy due to its high efficacy and selectivity and lower side effects compared with traditional treatment. The identification of tumor T cell antigens is one of the most important tasks for antitumor vaccines development and molecular function investigation. Although several machine learning predictors have been developed to identify tumor T cell antigen, more accurate tumor T cell antigen identification by existing methodology is still challenging.

**Methods:**

In this study, we used a non-redundant dataset of 592 tumor T cell antigens (positive samples) and 393 tumor T cell antigens (negative samples). Four types feature encoding methods have been studied to build an efficient predictor, including amino acid composition, global protein sequence descriptors and grouped amino acid and peptide composition. To improve the feature representation ability of the hybrid features, we further employed a two-step feature selection technique to search for the optimal feature subset. The final prediction model was constructed using random forest algorithm.

**Results:**

Finally, the top 263 informative features were selected to train the random forest classifier for detecting tumor T cell antigen peptides. iTTCA-RF provides satisfactory performance, with balanced accuracy, specificity and sensitivity values of 83.71%, 78.73% and 88.69% over tenfold cross-validation as well as 73.14%, 62.67% and 83.61% over independent tests, respectively. The online prediction server was freely accessible at http://lab.malab.cn/~acy/iTTCA.

**Conclusions:**

We have proven that the proposed predictor iTTCA-RF is superior to the other latest models, and will hopefully become an effective and useful tool for identifying tumor T cell antigens presented in the context of major histocompatibility complex class I.

**Supplementary Information:**

The online version contains supplementary material available at 10.1186/s12967-021-03084-x.

## Introduction

According to a report from the International Agency for Research on Cancer (IARC), approximately 10 million people die of cancer, and there were 19.3 million new cancer cases worldwide in 2020. Cancer has become the second leading cause of death [1, 2]. Tumor molecular targeted therapy, radiotherapy and chemotherapy together constitute the main means of modern cancer drug therapy [3–6]. Classic broad-spectrum anticancer drugs and radiotherapy are lethal to tumor cells, but they can destroy normal cells in the body, produce large adverse reactions, and are prone to drug resistance [7–11]. Advances in equipment and immuno-oncology are driving a revolution in the field of cancer care. New cancer treatments are emerging, and targeted immunotherapy is one of the most promising treatment options. Unlike the harmful side effects of chemotherapy and radiotherapy, immunotherapy has been proven to be highly selective and effective, while also reducing side effects [12–15]. Immunotherapy provides new opportunities for the development of potential cancer treatments. T cells can recognize and kill tumor antigens encountered on the surface, which are presented by major histocompatibility complex (MHC) class I and class II molecules on the antigen-presenting cell surface [16–18]. Therefore, T cells play an important role in the field of tumor rejection and immunotherapeutic cancer. Correctly identifying T cell antigens not only helps to understand their protective mechanism but also contributes to the development of highly efficient cancer peptide vaccines [19].

Although the experimental methods are considered to be the most reliable method to characterize the biological activity of T cell epitopes in tumor antigens, they are usually time-consuming and expensive. Due to their convenience and high efficiency, computational methods have attracted increasing attention in the field of bioinformatics [20–30]. In this study, we focused on the identification of tumor T cell antigens (TTCAs) represented by MHC class I. According to our research, only two machine learning prediction tools have been published to identify this type of TTCA. The first prediction model was introduced by Lissabet et al. and is called TTAgP1.0 [31]. TTAgP1.0 uses the relative frequency of amino acids and amino acid composition (AAC) to encode the peptide sequences and then employs random forest (RF) classifier to build the prediction model [32]. Regrettably, TTAgP1.0 neither provides a web-server nor the dataset used. Therefore, its usage for the related research community is quite limited, although it has its own advantages and reasonable prediction accuracy. Very recently, Charoenkwan et al. proposed another random forest based prediction model iTTCA-Hybrid [33]. Five feature extraction methods, namely, AAC, pseudo amino acid composition (PAAC), dipeptide composition (DPC), amino acid property distribution (CTDD) and physicochemical (PCP), were investigated. The final model was constructed using the hybrid features of PAAC and CTDD. In addition, the oversampling technique was also applied to address the problem of data imbalance.

**Fig. 1:**
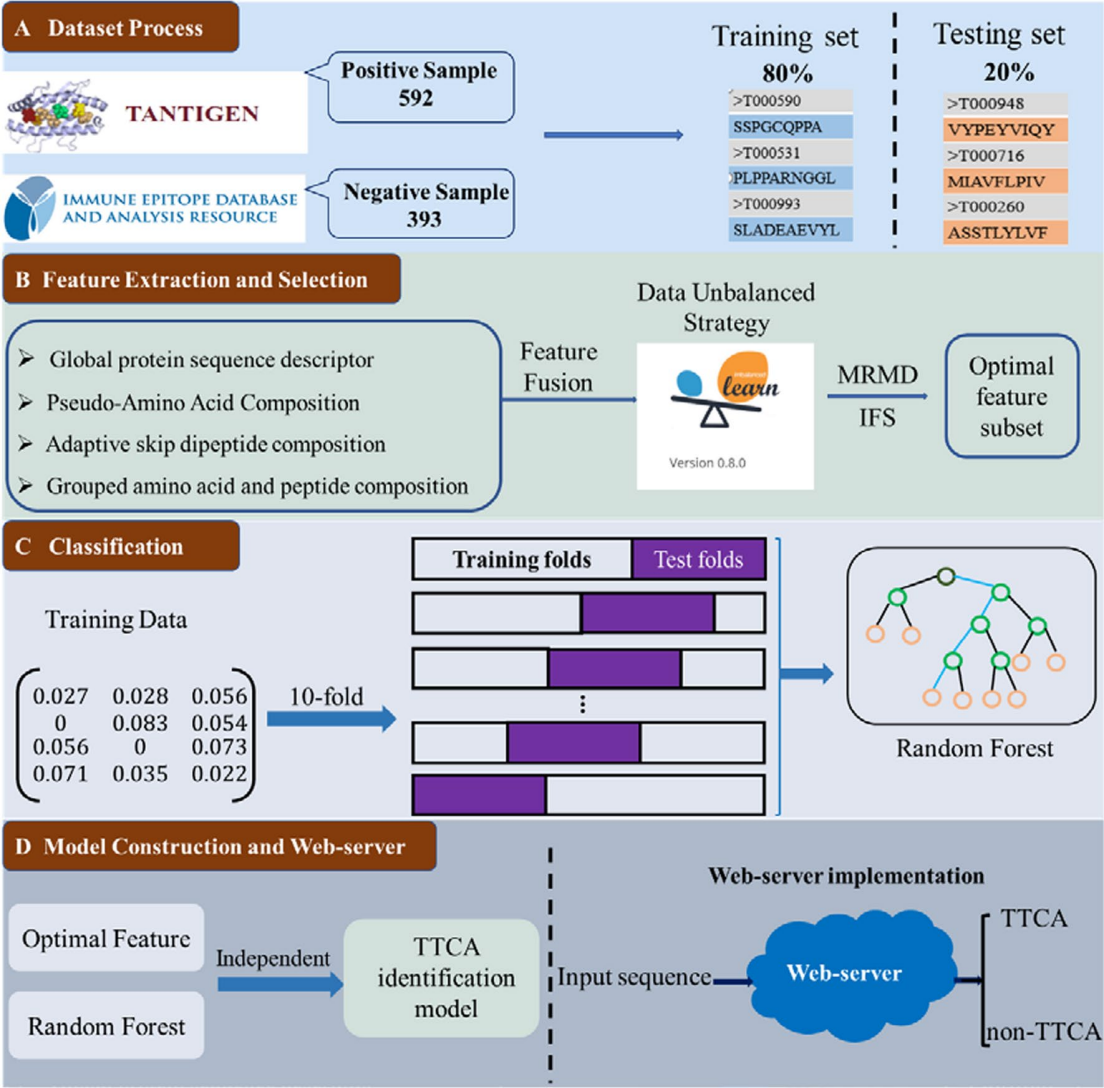
The whole framework of the proposed method iTTCT-RF to identify tumor T cell antigen

In this paper, we present a new predictor, iTTCA-RF, to distinguish TTCA from non-TTCA more accurately. As shown in Fig. 1, the protein sequences were preliminarily encoded using four kinds of feature extraction methods, namely global protein sequence descriptors (GPSD), grouped amino acid and peptide composition (GAAPC), PAAC and adaptive skip dipeptide composition (ASDC). We have investigated the performance of four single descriptors and their all possible combinations on six commonly classifiers, where the imbalanced training samples were handled by the hybrid-sampling approach SMOTE-Tomek. The results suggest that the hybrid feature composed of GPSD, GAAC and PAAC was the most informative for TTCA identification. Then, the maximum relevance maximum distance (MRMD) algorithm was used to analyze the feature importance of the involved vectors. With the application of the incremental feature selection (IFS) strategy, different feature subsets are generated for optimization under consideration of the classification algorithms. Ultimately, the best performance model was finally constructed using the top 263 selected features. The tenfold cross-validation (CV) scores of iTTCA-RF were balanced accuracy (BACC) = 83.71%, MCC = 0.678, AUC = 0.894, Sn = 88.69% and Sp = 78.73%, while those of the latest iTTCA-Hybrid were BACC = 78.83%, MCC = 0.588, AUC = 0.840, Sn = 85.53%, Sp = 72.13%. The iTTCA-RF achieved scores with BACC = 73.14%, MCC = 0.474 and Sp = 62.67% over the independent test, which means relative improvements of 2.4%, 4.6% and 4.0%, respectively, compared to the existing state-of-the-art model. We also established a user-friendly web server, which is expected to be an effective and useful tool for TTCA identification.

## Materials and methods

### Datasets

In this research, we directly used the benchmark datasets collected by Charoenkwan et al. [33]. The dataset was constructed as follows: (1) a total of 727 MHC class I peptides were collected as positive samples from TANTIGEN [34] and TANTIGEN 2.0 [12]; (2) non-TTCA was collected from the IEDB database [35], in addition, samples with no relationship with any disease were chosen as negative samples; and (3) duplicate peptide sequences were eliminated. Ultimately, 592 positive and 393 negative samples were obtained. As shown in Table 1, 80% of the samples were randomly selected as the training dataset and while the remaining 20% of samples as the independent test datasets.

**Table 1:** Sample distribution in the training and independent test datasets

Dataset	Training	Testing
Positive	470	122
Negative	318	75

### Feature representation

The quality of extracted sample features will greatly affect the performance of the predictive model. Researchers have proposed various biological sequence encoding strategies that can conveniently convert protein sequences into numerical vectors [22, 36–46]. In this paper, four feature encoding methods described below were adopted to represent the peptide sequences. In this work, we used the iLearn tool package [37] to generate the four type of sequence features.

#### Global protein sequence descriptor (GPSD)

This method (also called 188D features in many studies) describes the global composition of amino acid properties in a protein sequence and generates 188 features that integrate both sequence information and amino acid properties [47, 48]. In general, the GPSD descriptor contains two parts. The first part is the amino acid composition. The amino acid frequency in the peptide was calculated to obtain the first 20 features. The second part is the 168 features related to eight physicochemical properties of amino acids. Detailed information about the eight physicochemical properties of amino acids is described in the References [49–51]. For each property, 20 amino acids were divided into three groups, and the CTD (C: composition, T: transition and D: distribution) pattern was applied to encode the peptide sequences to generate 21D features. C stands for the occurrence frequencies of each group (3D). T represents the transition frequencies between the three groups (3D). D refers to the first, 25%, 50%, 75% and last occurring positions of a certain group in the peptide sequence (5 × 3 = 15D). Thus, the CTD model will produce 8 * (3 + 3 + 15) = 168 features.

#### Grouped amino acid and peptide composition (GAAPC)

According to their physical–chemical properties (e.g. molecular size, hydrophobicity and charge), the 20 amino acids (AAs) are further divided into five categories. The five categories are aliphatic group c1: GAVLMI, aromatic group c2: FYW, positively charged groups c3: KRH, negatively charged group c4: DE, and uncharged group c5: STCPNQ. A protein sequence of length L, can be coded as follows.

The grouped amino acid composition (GAAC) [37, 52] descriptor can be defined as:

1 $$ {\text{f}}\left( {\text{c}} \right) = \frac{{{\text{N}}\left( {\text{c}} \right)}}{{\text{L}}};{\text{~y}} \in \left\{ {{\text{c}}1,{\text{c}}2,{\text{c}}3,{\text{c}}4,{\text{c}}5} \right\};{\text{~}} $$

2 $$ {\text{N}}\left( {{\text{c}}_{{\text{i}}} } \right) = \sum {\text{N}}\left( {\text{i}} \right),\quad {\text{i}} \in {\text{c}} $$

where N(c) represents the number of AAs in group c, and N(i) is the number of AAs of type i.

The grouped dipeptide composition (GDPC) [37, 52] encoding is also a variation of the dipeptide composition descriptor. It is defined as:

3 $$ {\text{f}}\left( {{\text{x}},{\text{y}}} \right) = \frac{{{\text{N}}_{{{\text{xy}}}} }}{{{\text{L}} - 1}},\quad{\text{x}},{\text{y}} \in \left\{ {{\text{c}}1,{\text{c}}2,{\text{c}}3,{\text{c}}4,{\text{c}}5} \right\}; $$

where N_xy_ is the number of dipeptides represented by AA type groups x and y.

The grouped tripeptide composition (GTPC) [37, 52]encoding is a variation of the tripeptide composition descriptor. It is defined as:

4 $$ {\text{f}}\left( {{\text{x}},{\text{y}},{\text{z}}} \right) = \frac{{{\text{N}}_{{{\text{xyz}}}} }}{{{\text{L}} - 2}}{\text{~~~x}},{\text{y}},{\text{z}} \in \left\{ {{\text{c}}1,{\text{c}}2,{\text{c}}3,{\text{c}}4,{\text{c}}5} \right\} $$

where N_xyz_ refers to the number of tripeptides represented by AA type groups x, y and z.

GAAPC is a combination of GAAC, GDPC and GTPC. This method will produce 155D feature vectors.

#### Adaptive skip dipeptide composition (ASDC)

The ASDC descriptor was first presented by Wei et al. [53]. This method is another variant dipeptide composition that considers not only the relevant information between adjacent residues, but also that of intervening residues [54, 55]. It is defined as:

5 $$ {\text{ASDC}} = \left( {FV_{1} ;FV_{2} ; \ldots ;FV_{{400}} } \right) $$

6 $$ {\text{F}}V_{i}  = \frac{{\mathop \sum \nolimits_{{k = 1}}^{{N - 1}} f_{i}^{k} }}{{\mathop \sum \nolimits_{{i = 1}}^{{400}} \mathop \sum \nolimits_{{k = 1}}^{{N - 1}} f_{i}^{k} }} $$

where $\mathrm{F}\mathrm{V}\mathrm{i}$ represents the occurrence frequency of all possible dipeptides with ≤ N-1 intervening amino acids. In the ASDC method, the sequence can be easily converted to a 400-dimensional vector.

#### Pseudo-amino acid composition (PAAC)

The PAAC descriptor is a very effective feature extraction method and is widely used in protein attribute prediction, drug development and studies on drug target areas [56]. The sequence order correlation factors in PAAC incorporate the sequence-order information to some extent. Additional details of the PAAC features are described in the References [56–59]. We used the default parameters in iLearn to obtained a 22-dimensional feature vector.

### Classifiers

Six widely used classifiers were investigated to search for the most suitable machine learning algorithm, including random forest (RF), support vector machine (SVM), adaboost (AB), logistic regression (LR), bagging and gradient boosting machine (GBM). These efficient classification models in the scikit-learn package [60] were applied for models implementation and feature importance analysis. The hyper-parameters were optimized using grid search, and the search range was presented in Additional file 1: Table S1.

### Feature selection

The features extracted from a sequence in machine learning modeling always contain noise. To improve the feature representation ability, feature selection strategies are often adopted to solve the problems of redundant information and overfitting. Various approaches have been developed to analyze the features, such as analysis of variance (ANOVA) [61–65], minimal redundancy-maximal relevance (MRMR) [66–68] and MRMD [69–72]. These methods have been widely used in the field of RNA, DNA and protein prediction. In this work, MRMD was used to select optimal features for model training. The MRMD feature selection method is mainly determined by two parts [73]. The first part is the correlation between the feature and target class vector calculated by the Pearson correlation coefficient. The second part is the redundancy between features determined by three distance formulas (i.e., Euclidean distance, cosine distance and the Tanimoto coefficient). The larger the Pearson correlation coefficient is, the closer the relationship between the feature and the class label, and the larger the distance is, the lower the redundancy between the features. Finally, MRMD selects a subset of features that are strongly correlated with the class label and have low redundancy between features. We ranked the original features based on the MRMD feature sorting algorithm and then applied the IFS strategy to search for the optimal feature subset.

### Unbalanced strategy

Data imbalance has been encountered in multiple areas, such as bioinformatics, drug discovery, and disease diagnosis, and has been considered one of the top ten problems in pattern recognition and data mining [74–80]. Fortunately, several approaches have been specifically proposed by researchers to handle such datasets. The data level strategy is a direct way to balance the dataset by increasing/deleting the number of samples in the minority (majority) class. It can be divided into three categories, namely the over-sampling, under-sampling and hybrid-sampling methods [81–83]. In this research, we chose the hybrid-sampling method SMOTE-Tomek to balance the training dataset. This approach is a combination of over- and under-sampling methods: synthetic minority over-sampling technique (SMOTE) [84] and Tomek’s links (Tomek) [85]. This hybrid-sampling approach can simultaneously avoid the shortcomings of overfitting and loss of key information caused by SMOTE and Tomek, respectively.

### Evaluation parameters and strategies

According to previous related studies, there are three commonly used methods to evaluate the models in the field of protein prediction: K-fold CV, independent test and jackknife test. In this study, we used tenfold CV and independent tests to evaluate and optimize the model. For the binary classification, the confusion-matrix-based metrics are usually applied to measure the predictor, including accuracy (ACC), true negative rate (TNR)/specificity (Sp), true positive rate (TPR)/sensitivity (Sn), and matthew’s correlation coefficient (MCC) [86–101]. However, ACC does not perform well with imbalanced datasets, therefore, balanced accuracy (BACC) was used to measure how accurate is the overall performance of the models in this work. The formulas for these metrics are presented below:

7 $$ \left\{ {\begin{array}{*{20}l}    {\begin{array}{*{20}c}    {{\text{Sn}},{\text{~~TPR}} = \frac{{{\text{TP}}}}{{{\text{TP}} + {\text{FN}}}}}  \\    {\begin{array}{*{20}c}    {{\text{Sp}},{\text{~~TNR}} = \frac{{{\text{TN}}}}{{{\text{TN}} + {\text{FP}}}}}  \\    {{\text{BACC}} = \frac{1}{2} \times \left( {{\text{TPR}} + {\text{TNR}}} \right)}  \\   \end{array} }  \\   \end{array} }  \\    {{\text{MCC}} = {\text{~}}\frac{{\left( {TP \times TN} \right) - \left( {FP \times FN} \right)}}{{\sqrt {\left( {{\text{TP}} + {\text{FP}}} \right) \times \left( {{\text{TP}} + {\text{FN}}} \right) \times \left( {{\text{TN}} + {\text{FP}}} \right) \times \left( {{\text{TN}} + {\text{FN}}} \right)} }}}  \\   \end{array} } \right. $$

where ${\text{TP}},\;{\text{TN}},\;{\text{FP}}\;{\text{and}}\;{\text{FN}}$ represent true positive samples, true negative samples, false positive samples and false negative samples, respectively. In addition, the area under the receiver operating characteristic (auROC, also called AUC) curve is also employed, which is used to illustrate the prediction performance of the proposed models.

## Results and discussion

### Performance of individual feature descriptor

First, we studied the performance of four feature representation methods on six widely used machine learning classifiers. The tenfold CV was used to evaluate all models for fair comparison. The corresponding experimental results were summarized in Additional file 1: Table S2 and the BACC scores were shown in Table 2. As shown in Table 2, the random forest algorithm had the highest BACC on the three features of GPSD, ASDC and GAAPC. Although the BACC of RF on PAAC was not the highest, it was only slightly lower than the highest LR. For the performance of the four feature coding methods on the RF classifier, GPSD had the highest BACC of 69.62%, followed by GAAPC and ASDC, which had BACC of 67.71% and 66.88%, respectively, and PAAC had the lowest BACC of 62.45%.

**Table 2:** Preliminary results of different feature descriptors using different classifiers

Features	Classifier (BACC%)					
	LR	Bagging	RF	AB	GBM	SVM
GPSD	63.09	68.51	**69.62**	67.51	65.60	65.81
ASDC	58.53	63.13	**66.88**	62.96	64.03	64.51
GAAPC	58.91	66.75	**67.71**	64.44	67.56	61.75
PAAC	66.01	61.79	62.45	62.68	62.34	**67.91**
GPSD_B^a^	67.12	75.23	**79.62**	73.54	76.91	76.13
ASDC_B^a^	69.50	72.77	**79.19**	71.90	77.02	77.89
GAAPC_B^a^	66.33	75.51	**79.14**	73.02	78.34	74.94
PAAC_B^a^	66.63	71.77	**77.46**	69.91	74.29	73.09

The best performance value is highlighted in bold for clarification

^a^SMOTE-Tomek technique was applied to balance the data set

As discussed earlier, in unbalanced prediction tasks, conventional classifiers usually show poor recognition ability on minority classes. To enhance the performance of these predictive models, we used integrated resampling technique SMOTE-Tomek to balance the positive and negative samples and the results were Additional file 1: Table S3 and the BACC scores were also presented in Table 2. For a more intuitive comparison, the ROC curves before and after resampling were plotted in Additional file 1: Figure S1–4. The performance of all models was significantly improved after using SMOTE-Tomek except for the PAAC on LR classifier. Obviously, RF classifier again top ranked for its high BACC and AUC values on all balanced data. Similar to the results before resampling, the GPSD on the RF classifier showed the best performance among all the feature descriptors with an BACC score of 79.62% and PAAC performed the worst with BACC of 77.46%.

### Performance of hybrid features

By using a combination of various feature types, each can alleviate the others’ weaknesses and can integrate more sequence information, which helps predict TTCA. Based on these facts, comprehensive prediction performances of hybrid features were further evaluated. We evaluated all possible 11 combinations of four single descriptors, where the imbalanced samples were handled by the SMOTE-Tomek. Using the 11 hybrid features and 6 classifiers, we re-constructed 66 predictive models and the tenfold CV results on training set were provided in Additional file 1: Table S3. As shown in Table S4, almost all hybrid features performed best on RF except for the combination of ASDC + PAAC. This confirmed once again that RF is the most suitable classifier to distinguish TTCA from non-TTCA. In order to find the best hybrid feature to construct the optimal prediction model, we presented the prediction results of RF classifier in Table 3. It can be clearly seen from Table 3 that all hybrid features perform better than the individual features except for ASDC + PAAC combination. There seems no distinct regularity between the combination manner and the performance of corresponding model. The GPSD + GAAPC + PAAC combination yield the best prediction capability among all features in four metrics, with BACC of 83.03%, Sn of 88.69%, Sp of 77.38% and MCC of 0.665. When further integrating ASDC, the overall performance of the model drops sharply, which may be caused by the redundant features introduced by ASDC. Combining the results in Table 2, we can conclude that those models containing GPSD information GPSD are better than those without GPSD information. This indicates that GPSD descriptor is more predictive and discriminative than the others for TTCA prediction. Altogether, the GPSD + GAAPC + PAAC combination outperforms all the features (including individual features and hybrid features), and was selected for the next feature analysis experiment.

**Table 3:** The classification results of different hybrid features, (1): GPSD, (2): ASDC, (3): GAAPC, (4): PAAC

feature	BACC (%)	AUC	Sn (%)	Sp (%)	MCC
(1) + (2)	80.67	0.875	87.87	73.48	0.620
(1) + (3)	81.26	0.880	86.68	75.85	0.629
(1) + (4)	80.54	0.870	86.20	74.89	0.615
(2) + (3)	81.05	0.863	87.89	74.22	0.627
(2) + (4)	79.01	0.870	83.30	74.73	0.582
(3) + (4)	79.91	0.860	84.28	75.55	0.601
(1) + (2) + (3)	81.12	**0.883**	88.54	73.71	0.629
(1) + (2) + (4)	80.11	0.876	86.74	73.48	0.608
(1) + (3) + (4)	**83.03**	0.882	**88.69**	**77.38**	**0.665**
(2) + (3) + (4)	80.79	0.874	86.24	75.33	0.619
(1) + (2) + (3) + (4)	80.97	0.878	86.71	75.23	0.623

The best performance value is highlighted in bold for clarification

### Performance of optimal feature subset

**Fig. 2:**
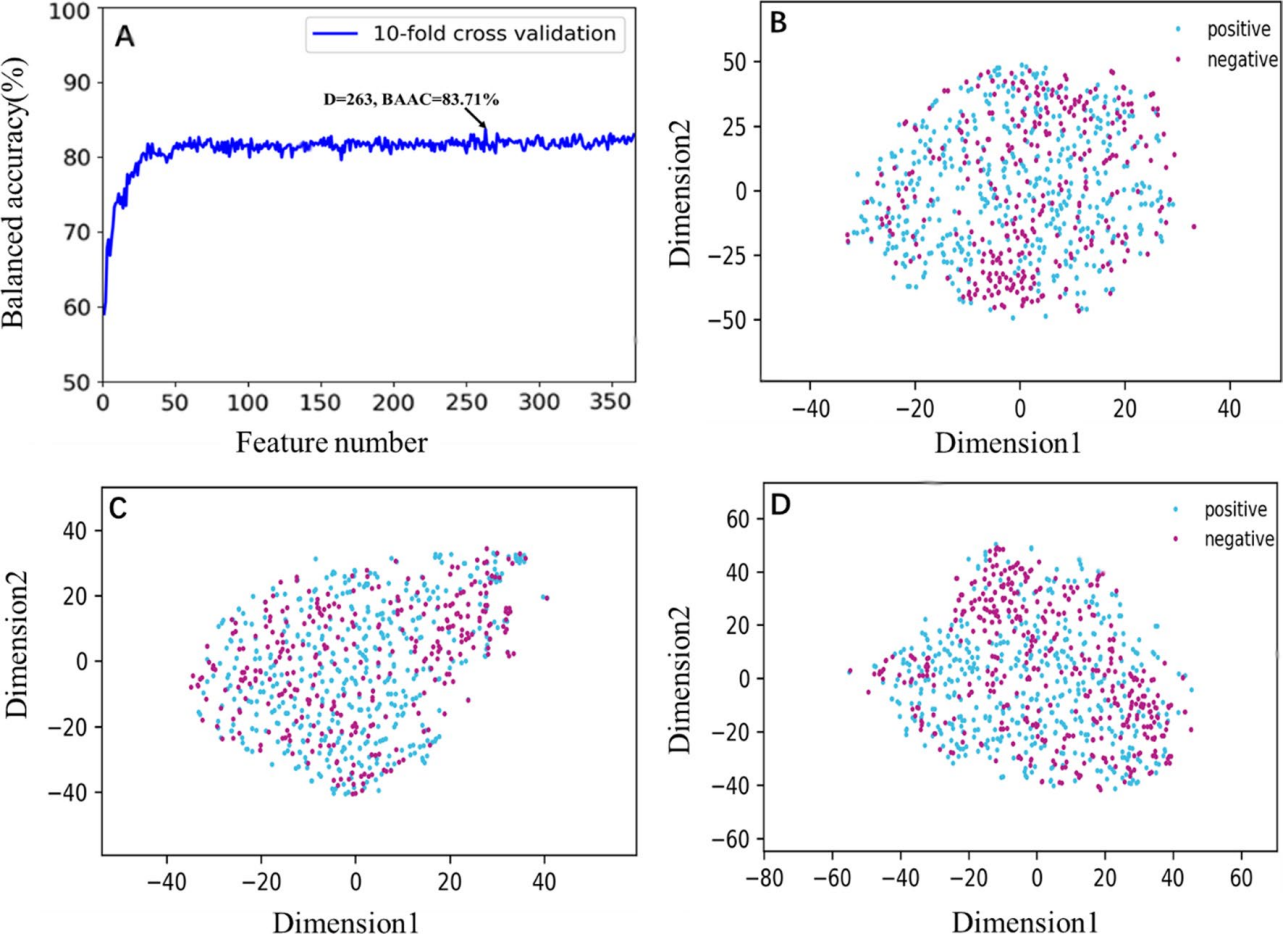
**A** Optimal feature subset selected by the MRMD and IFS strategy, tenfold CV BACC of the RF classifier with the feature number varied; **B** t-SNE distribution of the individual feature descriptor GPSD; **C** t-SNE distribution of the individual feature descriptor GAAPC; **D** t-SNE distribution of the optimal feature subset F263

To determine the optimal feature subset, we first sorted the original 365-dimensional hybrid feature (i.e. GPSD + GAAPC + PAAC obtained in “Performance of hybrid features” section) according to their importance measured by the MRMD algorithm. In the second step, the IFS strategy was applied to further determine the feature vector space for the RF classifier. A total of 365 RF models were trained on 365 feature subsets with 1, 2, 3…, 365 features. The five metrics mentioned above were used to evaluate the models. As shown in Fig. 2A, the tenfold CV BACC scores increased sharply as features were added when the dimension of the feature was less than 60, and then approached slowly fluctuating rising plateaus. When feature dimensions reached 263, the model achieved maximum tenfold CV BACC of 83.71% (Detailed results were presented in Table 4). To build a model with good robustness and generalization, the top 263-dimensional feature subset was selected as the final optimal feature space (named F263).

**Table 4:** Comparison of iTTCA-RF and state-of-the-art predictors

Tools	Tenfold CV					Independent test				
	BACC (%)	AUC	Sn (%)	Sp (%)	MCC	BACC (%)	AUC	Sn (%)	Sp (%)	MCC
TTAgP1.0	63.68	0.838	70.85	56.50	**0.838**	68.68	0.747	78.69	58.67	0.379
iTTCA-Hybrid	78.83	0.840	85.53	72.13	0.588	70.73	**0.783**	82.79	58.67	0.428
iTTCA-RF	**83.71**	**0.894**	**88.69**	**78.73**	0.678	**73.14**	0.780	**83.61**	**62.67**	**0.474**

The best performance value is highlighted in bold for clarification

Moreover, the extensively used data visualization method t-distributed stochastic neighbor embedding (t-SNE) [102]was utilized to validate the effective representation ability of the optimal feature set. We compared our optimal feature F263 with the two best performing individual feature descriptors (GPSD and GAAPC). The t-SNE were calculated for TTCA and non-TTCA of the three compared feature vectors and were plotted in Fig. 2B–D. For the original individual feature descriptors GPSD, the positive samples were randomly distributed in the feature space, while a small number of negative samples were concentrated in the upper and lower positions of the graph. As for GAAPC, most of positive and negative samples were randomly distributed and overlapping. For these two distribution maps, most samples overlap, and it was difficult to fit a boundary that distinguished the two types of samples. However, shown in Fig. 2D, although the distribution of positive samples and negative samples in the optimal F263 feature space still overlapped somewhat, it was simpler and clearer to find the dividing line that could distinguish most negative samples from positive samples. This indicates that using the 263-dimensional feature subset obtained of hybrid features by MRMD is easier to identify TTCA and non-TTCA samples than when using the original individual feature descriptors. Therefore, the tenfold CV results of iTTCA-RF were improved.

### Comparison with reported tools

Two classifiers to discriminate TTCA and non-TTCA have been published: TTAgP1.0 and iTTCA-Hybrid. Table 4 summarizes the tenfold CV and independent test scores of the three predictors. The results of TTAgP1.0 were from TTAgP1.0-MODI established by Charoenkwan et al. using the same method on the new dataset. Thus, all three tools were compared on the same training and testing dataset. Since almost all the results metrics of iTTCA-Hybrid were better than TTAgP1.0, we mainly compared iTTCA-RF with iTTCA-Hybrid.

**Fig. 3:**
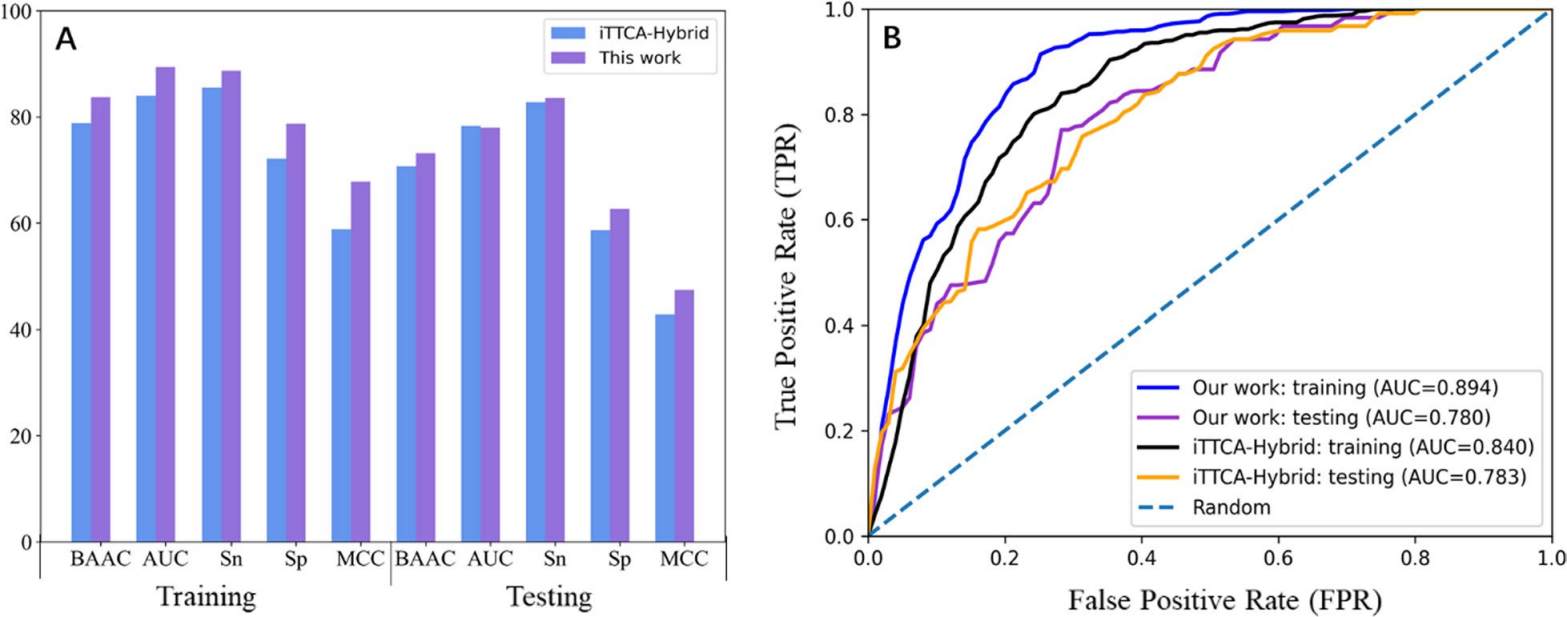
**A** Comparison of our results and the iTTCA-Hybrid predictor and **B** ROC curves of training and testing datasets curves for iTTCA-RF and iTTCA-Hybrid, respectively

Figure 3A also visually demonstrates the comparison of evaluation metrics, and ROC curves were drawn (Fig. 3B) to depict the prediction efficiency. As shown in Fig. 3, the tenfold CV of iTTCA-RF scores was higher than that of the iTTCA-Hybrid scores in almost all metrics. The BACC, AUC, Sn, Sp and MCC of our model on the training set were 4.9%, 5.4%, 3.2%, 6.6% and 9.0% higher than those of iTTCA-Hybrid, respectively. In terms of independent test scores, BACC, Sn, Sp and MCC outperformed iTTCA-Hybrid with improvement of 2.4%, 0.8%, 4.0% and 4.6%, respectively. These results indicate that the prediction capacity on negative samples of iTTCA-RF was greatly improved compared with the other predictors. Overall, iTTCA-RF significantly outperformed the other latest predictors, indicating that it can distinguish true TTCA from non-TTCA more accurately than existing tools. Although the developed predictor showed good performance, there is still much room for improvement, especially in terms of the predictive ability on negative samples.

### Web server implementation

For convenience, a user-friendly online server called iTTCA-RF was developed, which can be accessed freely at http://lab.malab.cn/~acy/iTTCA. Users can use the web-server to identify whether their protein sequences (in FASTA format) are TTCA or non-TTCA. The first step is to enter or paste the FASTA format protein sequences in the left blank box and then click the Submit button. The identification results will be displayed in the box on the right. If starting a new task, the user needs to click the Clear button or the Resubmit button to clear the input box. The Submit button will be reactivated, and the user will be allowed to input new query protein sequences. The homepage also provides links to download relevant data and contact the author.

## Conclusion

Accurate identification of TTCA will greatly promote cancer vaccine research and development. In this study, we constructed a new computational TTCA identifier named iTTCA-RF using the hybrid features of GPSD, GAAPC and PAAC. Combining the feature selection technique MRMD followed by IFS theory, the top 263 important features were chosen to build the best performance predictor. Here, the imbalance problem was addressed using the resampling method SMOTE-Tomek. iTTCA-RF achieves the best CV evaluation BACC value of 83.71%, which is 4.9% higher than the corresponding value of the previously reported best predictor. The independent test BACC score was 73.14%, an improvement of 2.4%, and associated Sp and MCC values were also increased by 4.0% and 4.6%, respectively. Meanwhile, a user-friendly web-server was also established. It is expected that iTTCA-RF will be a robust, reliable, and useful computational tool for tumor T cell antigen identification. Although our proposed model is superior to other published predictors, the model requires further development, especially the ability to identify negative samples. Future work will focus on exploring deep learning [103, 104] or more effective feature representation or computational intelligence strategies to improve the model’s performance.

## Supplementary Information

**Additional file 1: Table S1.** Hyperparameters search range for Random Forest classifier. **Table S2.** Results of single feature descriptors without SMOTE-Tomek over 10-fold CV. **Table S3.** Results of single feature descriptor with SMOTE-Tomek over 10-fold CV. **Table S4.** Results of hybrid feature descriptor with SMOTE-Tomek over 10-fold CV. **Figure S1.** 10-fold CV ROC curves for GPSD descriptor on six classifiers. **Figure S2.** 10-fold CV ROC curves for ASDC descriptor on six classifiers. **Figure S3.** 10-fold CV ROC curves for GAAPC descriptor on six classifiers. **Figure S4.** 10-fold CV ROC curves for PAAC descriptor on six classifiers.

## Data Availability

Publicly available datasets were analyzed in this study. This data can be found here: http://lab.malab.cn/~acy/iTTCA.

## Abbreviations

IARC: International Agency for Research on Cancer
MHC: Major histocompatibility complex
TTCA: Tumor T cell antigens
AAC: Amino acid composition
RF: Random Forest
PAAC: Pseudo amino acid composition
CTDD: Amino acid property distribution
GPSD: Global protein sequence descriptors
GAAPC: Grouped amino acid and peptide composition
ASDC: Adaptive skip dipeptide composition
MRMD: Maximum relevance maximum distance
IFS: Incremental feature selection
CV: Cross-validation
BACC: Balanced accuracy
AAs: Amino acids
GAAC: Grouped amino acid composition
GDPC: Grouped dipeptide composition (GDPC)
GTPC: Grouped tripeptide composition
SVM: Support vector machine
AB: Adaboost
LR: Logistic regression
GBM: Gradient boosting machine
ANOVA: Analysis of variance
SMOTE: Synthetic minority over-sampling technique
ACC: Accuracy
TNR: True negative rate
TPR: True positive rate
Sp: Specificity
Sn: Sensitivity
MCC: Matthew’s correlation coefficient
AUC: Area under the receiver operating characteristic curve

## References

[1] Zhang ZM (2020). Early diagnosis of pancreatic ductal adenocarcinoma by combining relative expression orderings with machine-learning method. Front Cell Dev Biol.

[2] Cheng L (2018). DincRNA: a comprehensive web-based bioinformatics toolkit for exploring disease associations and ncRNA function. Bioinformatics.

[3] Burugu S, Dancsok AR, Nielsen TO (2018). Emerging targets in cancer immunotherapy. Semin Cancer Biol.

[4] Dong Y-M (2020). ESDA: an improved approach to accurately identify human snoRNAs for precision cancer therapy. Curr Bioinform.

[5] Yu L (2021). Predicting therapeutic drugs for hepatocellular carcinoma based on tissue-specific pathways. PLoS Comput Biol.

[6] Behl T (2020). Gene therapy in the management of Parkinson's disease: potential of gdnf as a promising therapeutic strategy. Curr Gene Ther.

[7] Couzin-Frankel J (2013). Cancer immunotherapy. Science.

[8] Li Z (2020). Research on gastric cancer's drug-resistant gene regulatory network model. Curr Bioinform.

[9] Ding Y, Tang J, Guo F (2020). Identification of drug-target interactions via dual laplacian regularized least squares with multiple kernel fusion. Knowl Based Syst.

[10] Ding Y, Tang J, Guo F (2020). Identification of drug-target interactions via fuzzy bipartite local model. Neural Comput Appl.

[11] Ding Y, Tang J, Guo F (2017). Identification of drug-target interactions via multiple information integration. Inf Sci.

[12] Zhang G (2021). TANTIGEN 2.0: a knowledge base of tumor T cell antigens and epitopes. BMC Bioinform.

[13] Zhao X (2019). Predicting drug side effects with compact integration of heterogeneous networks. Curr Bioinform.

[14] Ding Y, Tang J, Guo F (2019). Identification of drug-side effect association via multiple information integration with centered kernel alignment. Neurocomputing.

[15] Shang Y (2021). Prediction of drug-target interactions based on multi-layer network representation learning. Neurocomputing.

[16] Aranda F (2013). Trial watch peptide vaccines in cancer therapy. Oncoimmunology.

[17] Liu Y (2020). A review on the methods of peptide-MHC binding prediction. Curr Bioinform.

[18] Wang P (2020). Comprehensive analysis of TCR repertoire in COVID-19 using single cell sequencing. Genomics.

[19] Ren X (2021). COVID-19 immune features revealed by a large-scale single-cell transcriptome atlas. Cell.

[20] Liu K, Chen W (2020). iMRM: a platform for simultaneously identifying multiple kinds of RNA modifications. Bioinformatics.

[21] Ao C, Yu L, Zou Q (2021). Prediction of bio-sequence modifications and the associations with diseases. Brief Funct Genomics.

[22] Liu B, Gao X, Zhang H (2019). BioSeq-Analysis2.0: an updated platform for analyzing DNA, RNA and protein sequences at sequence level and residue level based on machine learning approaches. Nucleic Acids Res.

[23] Zulfiqar H (2021). Screening of prospective plant compounds as H1R and CL1R inhibitors and its antiallergic efficacy through molecular docking approach. Comput Math Methods Med.

[24] Yang H (2021). Risk prediction of diabetes: big data mining with fusion of multifarious physical examination indicators. Inf Fus.

[25] Yu L, Shi Y, Zou Q, Wang S, Zheng L, Gao L (2020). Exploring drug treatment patterns based on the action of drug and multilayer network model. Int J Mol Sci.

[26] Fu X (2020). StackCPPred: a stacking and pairwise energy content-based prediction of cell-penetrating peptides and their uptake efficiency. Bioinformatics.

[27] Zeng X (2020). Target identification among known drugs by deep learning from heterogeneous networks. Chem Sci.

[28] Zeng X (2017). Prediction and validation of disease genes using HeteSim scores. IEEE/ACM Trans Comput Biol Bioinf.

[29] Cheng L (2019). MetSigDis: a manually curated resource for the metabolic signatures of diseases. Brief Bioinform.

[30] Hu Y (2021). rs1990622 variant associates with Alzheimer's disease and regulates TMEM106B expression in human brain tissues. BMC Med.

[31] Beltran Lissabet JF, Herrera Belen L, Farias JG (2019). TTAgP 10: a computational tool for the specific prediction of tumor T cell antigens. Comput Biol Chem.

[32] Ao C (2020). Prediction of antioxidant proteins using hybrid feature representation method and random forest. Genomics.

[33] Charoenkwan P (2020). iTTCA-Hybrid: Improved and robust identification of tumor T cell antigens by utilizing hybrid feature representation. Anal Biochem.

[34] Olsen LR (2017). TANTIGEN: a comprehensive database of tumor T cell antigens. Cancer Immunol Immunother.

[35] Vita R (2019). The immune epitope database (IEDB): 2018 update. Nucleic Acids Res.

[36] Muhammod R (2019). PyFeat: a Python-based effective feature generation tool for DNA, RNA and protein sequences. Bioinformatics.

[37] Chen Z (2020). iLearn: an integrated platform and meta-learner for feature engineering, machine-learning analysis and modeling of DNA, RNA and protein sequence data. Brief Bioinform.

[38] Wang H (2020). Identification of membrane protein types via multivariate information fusion with Hilbert-Schmidt Independence Criterion. Neurocomputing.

[39] Li J (2020). DeepAVP: a dual-channel deep neural network for identifying variable-length antiviral peptides. IEEE J Biomed Health Inform.

[40] Shen Y, Tang J, Guo F (2019). Identification of protein subcellular localization via integrating evolutionary and physicochemical information into Chou’s general PseAAC. J Theor Biol.

[41] Shen Y (2019). Critical evaluation of web-based prediction tools for human protein subcellular localization. Brief Bioinform.

[42] Tang Y-J, Pang Y-H, Liu B (2020). IDP-Seq2Seq: identification of intrinsically disordered regions based on sequence to sequence learning. Bioinformaitcs.

[43] Shao J, Yan K, Liu B (2021). FoldRec-C2C: protein fold recognition by combining cluster-to-cluster model and protein similarity network. Brief Bioinform.

[44] Cai L (2020). ITP-Pred: an interpretable method for predicting, therapeutic peptides with fused features low-dimension representation. Brief Bioinform.

[45] Jin S (2020). Application of deep learning methods in biological networks. Brief Bioinform.

[46] Zhao T (2020). DeepLGP: a novel deep learning method for prioritizing lncRNA target genes. Bioinformatics.

[47] Dubchak I (1995). Prediction of protein-folding class using global description of amino-acid-sequence. Proc Natl Acad Sci USA.

[48] Zou Q (2013). An approach for identifying cytokines based on a novel ensemble classifier. Biomed Res Int.

[49] Li Y, Niu M, Zou Q (2019). ELM-MHC: an improved MHC identification method with extreme learning machine algorithm. J Proteome Res.

[50] Xuan JJ (2018). RMBase v2.0: deciphering the map of RNA modifications from epitranscriptome sequencing data. Nucleic Acids Res.

[51] Lin C-W (2013). Kaempferol reduces matrix metalloproteinase-2 expression by down-regulating ERK1/2 and the activator protein-1 signaling pathways in oral cancer cells. PLoS ONE.

[52] Chen Z (2018). iFeature: a Python package and web server for features extraction and selection from protein and peptide sequences. Bioinformatics.

[53] Wei L, Tang J, Zou Q (2017). SkipCPP-Pred: an improved and promising sequence-based predictor for predicting cell-penetrating peptides. BMC Genom.

[54] Wei L (2018). ACPred-FL: a sequence-based predictor using effective feature representation to improve the prediction of anti-cancer peptides. Bioinformatics.

[55] Zhang D (2021). iBLP: an XGBoost-based predictor for identifying bioluminescent proteins. Comput Math Methods Med.

[56] Xu L (2018). A novel hybrid sequence-based model for identifying anticancer peptides. Genes.

[57] Chou K-C (2011). Some remarks on protein attribute prediction and pseudo amino acid composition. J Theor Biol.

[58] Chou KC (2005). Using amphiphilic pseudo amino acid composition to predict enzyme subfamily classes. Bioinformatics.

[59] Liu B, Zhu Y, Yan K (2020). Fold-LTR-TCP: protein fold recognition based on triadic closure principle. Brief Bioinform.

[60] Pedregosa F (2011). Scikit-learn: machine learning in python. J Mach Learn Res.

[61] Blanca MJ (2017). Non-normal data: is ANOVA still a valid option?. Psicothema.

[62] Tang H, Chen W, Lin H (2016). Identification of immunoglobulins using Chou's pseudo amino acid composition with feature selection technique. Mol BioSyst.

[63] Jung Y, Zhang H, Hu J (2019). Transformed low-rank ANOVA models for high-dimensional variable selection. Stat Methods Med Res.

[64] Tan JX (2019). Identification of hormone binding proteins based on machine learning methods. Math Biosci Eng.

[65] Han X (2021). SubtypeDrug: a software package for prioritization of candidate cancer subtype-specific drugs. Bioinformatics.

[66] Ju Z, Wang S-Y (2019). iLys-Khib: identify lysine 2-Hydroxyisobutyrylation sites using mRMR feature selection and fuzzy SVM algorithm. Chemom Intell Lab Syst.

[67] Mostafa SS, Morgado-Dias F, Ravelo-Garcia AG (2020). Comparison of SFS and mRMR for oximetry feature selection in obstructive sleep apnea detection. Neural Comput Appl.

[68] Wang J, Zhang D, Li J (2013). PREAL: prediction of allergenic protein by maximum Relevance Minimum Redundancy (mRMR) feature selection. BMC Syst Biol.

[69] Meng C (2020). CWLy-pred: a novel cell wall lytic enzyme identifier based on an improved MRMD feature selection method. Genomics.

[70] Tao Z (2020). A method for identifying vesicle transport proteins based on LibSVM and MRMD. Comput Math Methods Med.

[71] He S (2020). MRMD2.0: a python tool for machine learning with feature ranking and reduction. Curr Bioinform.

[72] Lu XX, Zhao SZ (2019). Gene-based therapeutic tools in the treatment of cornea disease. Curr Gene Ther.

[73] Zou Q (2016). A novel features ranking metric with application to scalable visual and bioinformatics data classification. Neurocomputing.

[74] Lemaitre G, Nogueira F, Aridas CK (2017). Imbalanced-learn: a python toolbox to tackle the curse of imbalanced datasets in machine learning. J Mach Learn Res.

[75] Yang X-F (2020). Predicting LncRNA subcellular localization using unbalanced pseudo-k nucleotide compositions. Curr Bioinform.

[76] Hasan MAM (2020). Citrullination site prediction by incorporating sequence coupled effects into PseAAC and resolving data imbalance issue. Curr Bioinform.

[77] Chao L, Wei L, Zou Q (2019). SecProMTB: a SVM-based classifier for secretory proteins of *Mycobacterium tuberculosis* with imbalanced data set. Proteomics.

[78] Yu L (2020). Prediction of drug response in multilayer networks based on fusion of multiomics data. Methods (San Diego, Calif.).

[79] Zeng X (2017). A comprehensive overview and evaluation of circular RNA detection tools. Plos Comput Biol.

[80] Zeng X (2018). Prediction of potential disease-associated microRNAs using structural perturbation method. Bioinformatics.

[81] Kaur H, Pannu HS, Malhi AK (2019). A systematic review on imbalanced data challenges in machine learning: applications and solutions. ACM Comput Surv.

[82] Branco P, Torgo L, Ribeiro RP (2016). A survey of predictive modeling on IM balanced domains. ACM Comput Surv.

[83] Zou Q (2016). Finding the best classification threshold in imbalanced classification. Big Data Res.

[84] Chawla NV (2002). SMOTE: synthetic minority over-sampling technique. J Artif Intell Res.

[85] Tomek I (1976). Two modifications of CNN. IEEE Trans Syst Man Cybern.

[86] Wang H, Tang J, Ding Y, Guo F (2021). Exploring associations of non-coding RNAs in human diseases via three-matrix factorization with hypergraph-regular terms on center kernel alignment. Brief Bioinform.

[87] Li J, Pu Y, Tang J, Zou Q, Guo F (2020). DeepATT: a hybrid category attention neural network for identifying functional effects of DNA sequences. Brief Bioinform.

[88] Hong Z (2020). Identifying enhancer-promoter interactions with neural network based on pre-trained DNA vectors and attention mechanism. Bioinformatics.

[89] Jin Q (2019). DUNet: a deformable network for retinal vessel segmentation. Knowl-Based Syst.

[90] Su R (2020). Empirical comparison and analysis of web-based cell-penetrating peptide prediction tools. Brief Bioinform.

[91] Wei L, Chen H, Su R (2018). M6APred-EL: a sequence-based predictor for identifying n6-methyladenosine sites using ensemble learning. Mol Ther Nucleic Acids.

[92] Wei L (2020). Computational prediction and interpretation of cell-specific replication origin sites from multiple eukaryotes by exploiting stacking framework. Brief Bioinform.

[93] Wei L (2020). Comparative analysis and prediction of quorum-sensing peptides using feature representation learning and machine learning algorithms. Brief Bioinform.

[94] Wei L (2014). Improved and promising identification of human MicroRNAs by incorporating a high-quality negative set. IEEE/ACM Trans Comput Biol Bioinf.

[95] Wei L (2017). A novel hierarchical selective ensemble classifier with bioinformatics application. Artif Intell Med.

[96] Wei L (2017). Improved prediction of protein-protein interactions using novel negative samples, features, and an ensemble classifier. Artif Intell Med.

[97] Shao J, Liu B (2021). ProtFold-DFG: protein fold recognition by combining directed fusion graph and PageRank algorithm. Brief Bioinform.

[98] Jiang Q (2013). Predicting human microRNA-disease associations based on support vector machine. Int J Data Min Bioinform.

[99] Yu L, Xu F, Gao L (2020). Predict new therapeutic drugs for hepatocellular carcinoma based on gene mutation and expression. Front Bioeng Biotechnol.

[100] Zeng X (2019). deepDR: a network-based deep learning approach to in silico drug repositioning. Bioinformatics.

[101] Hong Z (2019). Identifying enhancer–promoter interactions with neural network based on pre-trained DNA vectors and attention mechanism. Bioinformatics.

[102] van der Maaten L, Hinton G (2008). Visualizing data using t-SNE. J Mach Learn Res.

[103] Lv H (2020). Deep-Kcr: accurate detection of lysine crotonylation sites using deep learning method. Brief Bioinform.

[104] Dao FY (2020). DeepYY1: a deep learning approach to identify YY1-mediated chromatin loops. Brief Bioinform.

