# Supplementary material for: iTTCA-RF: a random forest predictor for tumor T cell antigens
Source: J Transl Med. 2021 Oct 27;19:449. doi: 10.1186/s12967-021-03084-x (PMC8554859; doi:10.1186/s12967-021-03084-x)
Supplement: Supplementary file 1 — Additional file 1: Table S1. Hyperparameters search range for Random Forest classifier. Table S2. Results of single feature descriptors without SMOTE-Tomek over 10-fold CV. Table S3. Results of single feature descriptor with SMOTE-Tomek over 10-fold CV. Table S4. Results of hybrid feature descriptor with SMOTE-Tomek over 10-fold CV. Figure S1. 10-fold CV ROC curves for GPSD descriptor on six classifiers. Figure S2. 10-fold CV ROC curves for ASDC descriptor on six classifiers. Figure S3. 10-fold CV ROC curves for GAAPC descriptor on six classifiers. Figure S4. 10-fold CV ROC curves for PAAC descriptor on six classifiers. [file 12967_2021_3084_MOESM1_ESM.docx]

**iTTCA-RF: A random forest predictor for tumor T cell antigens**

Shihu Jiao^1^, Quan Zou^1,2^, Huannan Guo^3,*^, Lei Shi^4,*^

1. Yangtze Delta Region Institute (Quzhou), University of Electronic Science and Technology of China, Quzhou, China
2. Institute of Fundamental and Frontier Sciences, University of Electronic Science and Technology of China, Chengdu, China
3. Department of Oncology, General Hospital of Heilongjiang Province Land Reclamation Bureau, Harbin, China
4. Department of Spine Surgery, Changzheng Hospital, Naval Medical University, Shanghai, China

*corresponding author: guohuannan1226@sohu.com & slspine@163.com

**Contents**

1. Additiona Tables

Table S1. Hyperparameters search range for Random Forest classifier. P2

Table S2 Results of single feature descriptors without SMOTE-Tomek over 10-fold CV. P2

Table S3 Results of single feature descriptor with SMOTE-Tomek over 10-fold CV. P3

Table S4 Results of hybrid feature descriptor with SMOTE-Tomek over 10-fold CV.

P3-P4

1. Additional Figures

Figure S1 10-fold CV ROC curves for GPSD descriptor on six classifiers P5

Figure S2 10-fold CV ROC curves for ASDC descriptor on six classifiers P5

Figure S3 10-fold CV ROC curves for GAAPC descriptor on six classifiers P5

Figure S4 10-fold CV ROC curves for PAAC descriptor on six classifiers P6

1. **Additional Tables**

**Table S1**. Hyperparameters search range for classifiers.

| Method | Parameter* | Tested values |
| --- | --- | --- |
| Logistic Regression (LR) | tol | 0.001-1 with an interval of 0.1 |
|  | C | 0.1-5 with an interval of 1 |
| Bagging | base_estimator | DecisionTreeClassifier |
|  | n_estimators | 20–200 with an interval of 20 |
| Random Forest (RF) | n_estimators | 20–200 with an interval of 20 |
|  | max_features | 1–20 with an interval of 2 |
| Adaboost (AB) | n_estimator | 20-200 with an interval of 20 |
|  | learning_rate | 0.001-0.5 with an interval of 0.1 |
| Gradient Boosting (GBM) | n_estimator | 20-200 with an interval of 20 |
|  | learning_rate | 0.001-0.5 with an interval of 0.1 |
| Support vector machine (SVM) | C | 2^-5^–2^15^ with step size of 2 |
|  | gamma | 2^-15^–2^-5^ with step size of 2^-1^ |

^*^Parameter name in the Scikit-learn implementation.

**Table S2** Results of single feature descriptors without SMOTE-Tomek over 10-fold CV.

| Feature descriptor | Classifier | BACC % | AUC | Sn % | Sp % | MCC |
| --- | --- | --- | --- | --- | --- | --- |
| GPSD | LR | 63.09 | 0.690 | 76.81 | 49.37 | 0.272 |
|  | Bagging | 68.51 | 0.733 | 79.79 | 57.23 | 0.380 |
|  | RF | **69.62** | 0.742 | 88.30 | 50.94 | **0.431** |
|  | AB | 67.51 | **0.747** | 78.72 | 56.29 | 0.359 |
|  | GBM | 65.60 | 0.728 | 83.40 | 47.80 | 0.337 |
|  | SVM | 65.81 | 0.714 | 90.43 | 41.19 | 0.372 |
| ASDC | LR | 58.53 | 0.629 | 62.98 | 54.09 | 0.169 |
|  | Bagging | 63.13 | 0.659 | 68.09 | **58.18** | 0.261 |
|  | RF | 66.88 | 0.736 | 86.60 | 47.17 | 0.373 |
|  | AB | 62.96 | 0.706 | 73.40 | 52.52 | 0.263 |
|  | GBM | 64.03 | 0.734 | 83.40 | 44.65 | 0.307 |
|  | SVM | 64.51 | 0.701 | 85.32 | 43.71 | 0.323 |
| GAAPC | LR | 58.91 | 0.635 | 71.91 | 45.91 | 0.183 |
|  | Bagging | 66.75 | 0.713 | 75.96 | 57.55 | 0.339 |
|  | RF | 67.71 | 0.728 | 84.47 | 50.94 | 0.380 |
|  | AB | 64.44 | 0.692 | 75.11 | 53.77 | 0.294 |
|  | GBM | 67.56 | 0.743 | 85.74 | 49.37 | 0.382 |
|  | SVM | 61.75 | 0.680 | 79.79 | 43.71 | 0.252 |
| PAAC | LR | 66.01 | 0.705 | 89.57 | 42.45 | 0.371 |
|  | Bagging | 61.79 | 0.648 | 70.43 | 53.14 | 0.237 |
|  | RF | 62.45 | 0.679 | 82.13 | 42.77 | 0.272 |
|  | AB | 62.68 | 0.679 | 78.51 | 46.86 | 0.267 |
|  | GBM | 62.34 | 0.673 | 81.91 | 42.77 | 0.270 |
|  | SVM | 67.91 | 0.741 | **90.85** | 44.97 | 0.414 |

**Table S3** Results of single feature descriptor with SMOTE-Tomek over 10-fold CV.

| Feature descriptor | Classifier | BACC % | AUC | Sn % | Sp% | MCC |
| --- | --- | --- | --- | --- | --- | --- |
| GPSD | LR | 67.12 | 0.757 | 67.34 | 66.89 | 0.342 |
|  | Bagging | 75.23 | 0.840 | 74.55 | 75.90 | 0.505 |
|  | RF | **79.62** | **0.865** | 86.94 | 72.30 | **0.599** |
|  | AB | 73.54 | 0.826 | 74.55 | 72.52 | 0.471 |
|  | GBM | 76.91 | 0.848 | 80.41 | 73.42 | 0.540 |
|  | SVM | 76.13 | 0.842 | 81.31 | 70.95 | 0.525 |
| ASDC | LR | 69.50 | 0.717 | 61.44 | **77.56** | 0.395 |
|  | Bagging | 72.77 | 0.804 | 71.24 | 74.29 | 0.456 |
|  | RF | 79.19 | 0.854 | **88.24** | 70.15 | 0.594 |
|  | AB | 71.90 | 0.801 | 74.29 | 69.50 | 0.438 |
|  | GBM | 77.02 | 0.846 | 82.79 | 71.24 | 0.544 |
|  | SVM | 77.89 | 0.851 | 80.61 | 75.16 | 0.559 |
| GAAPC | LR | 66.33 | 0.707 | 62.59 | 70.07 | 0.327 |
|  | Bagging | 75.51 | 0.828 | 74.83 | 76.19 | 0.510 |
|  | RF | 79.14 | 0.853 | 85.71 | 72.56 | 0.588 |
|  | AB | 73.02 | 0.825 | 74.15 | 71.88 | 0.460 |
|  | GBM | 78.34 | 0.849 | 83.67 | 73.02 | 0.570 |
|  | SVM | 74.94 | 0.819 | 74.38 | 75.51 | 0.499 |
| PAAC | LR | 66.63 | 0.729 | 74.40 | 58.86 | 0.337 |
|  | Bagging | 71.77 | 0.781 | 69.58 | 73.96 | 0.436 |
|  | RF | 77.46 | 0.844 | 83.15 | 71.77 | 0.553 |
|  | AB | 69.91 | 0.775 | 71.99 | 67.83 | 0.399 |
|  | GBM | 74.29 | 0.801 | 80.96 | 67.61 | 0.490 |
|  | SVM | 73.09 | 0.803 | 77.02 | 69.15 | 0.463 |

**Table S4** Results of hybrid features with SMOTE-Tomek over 10-fold CV.

| classifier | feature | BACC% | AUC | Sn% | Sp% | MCC | classifier | feature | BACC% | AUC | Sn% | Sp% | MCC |
| --- | --- | --- | --- | --- | --- | --- | --- | --- | --- | --- | --- | --- | --- |
| LR | (1)(2) | 76.52 | 0.819 | 70.34 | 82.70 | 0.534 | AB | (1)(2) | 76.85 | 0.833 | 77.75 | 75.96 | 0.537 |
|  | (1)(3) | 71.56 | 0.780 | 66.59 | 76.52 | 0.433 |  | (1)(3) | 75.62 | 0.832 | 74.94 | 76.30 | 0.512 |
|  | (1)(4) | 69.12 | 0.776 | 69.00 | 69.23 | 0.382 |  | (1)(4) | 73.87 | 0.815 | 75.57 | 72.17 | 0.478 |
|  | (2)(3) | 72.98 | 0.771 | 65.70 | 80.27 | 0.465 |  | (2)(3) | 71.64 | 0.807 | 70.40 | 72.87 | 0.433 |
|  | (2)(4) | 73.41 | 0.784 | 67.47 | 79.34 | 0.471 |  | (2)(4) | 73.96 | 0.797 | 73.41 | 74.51 | 0.479 |
|  | (3)(4) | 71.72 | 0.780 | 69.00 | 74.45 | 0.435 |  | (3)(4) | 74.34 | 0.801 | 75.55 | 73.14 | 0.487 |
|  | (1)(2)(3) | 77.19 | 0.825 | 71.01 | 83.37 | 0.548 |  | (1)(2)(3) | 74.94 | 0.816 | 75.73 | 74.16 | 0.499 |
|  | (1)(2)(4) | 77.30 | 0.826 | 71.46 | 83.15 | 0.550 |  | (1)(2)(4) | 72.70 | 0.817 | 72.13 | 73.26 | 0.454 |
|  | (1)(3)(4) | 72.62 | 0.793 | 68.10 | 77.15 | 0.454 |  | (1)(3)(4) | 75.11 | 0.832 | 76.24 | 73.98 | 0.502 |
|  | (2)(3)(4) | 74.67 | 0.801 | 68.12 | 81.22 | 0.498 |  | (2)(3)(4) | 74.24 | 0.823 | 74.45 | 74.02 | 0.485 |
|  | (1)(2)(3)(4) | 78.83 | 0.836 | 72.97 | **84.68** | 0.581 |  | (1)(2)(3)(4) | 75.11 | 0.815 | 75.68 | 74.55 | 0.502 |
| Bagging | (1)(2) | 77.53 | 0.845 | 75.96 | 79.10 | 0.551 | GBM | (1)(2) | 78.54 | 0.855 | 82.47 | 74.61 | 0.573 |
|  | (1)(3) | 78.67 | 0.856 | 76.75 | 80.59 | 0.574 |  | (1)(3) | 77.31 | 0.860 | 81.72 | 72.91 | 0.548 |
|  | (1)(4) | 76.81 | 0.848 | 75.57 | 78.05 | 0.536 |  | (1)(4) | 76.70 | 0.856 | 80.32 | 73.08 | 0.535 |
|  | (2)(3) | 75.11 | 0.827 | 73.09 | 77.13 | 0.503 |  | (2)(3) | 77.80 | 0.845 | 81.39 | 74.22 | 0.557 |
|  | (2)(4) | 74.95 | 0.826 | 71.43 | 78.46 | 0.500 |  | (2)(4) | 77.03 | 0.847 | 81.10 | 72.97 | 0.542 |
|  | (3)(4) | 74.78 | 0.819 | 74.02 | 75.55 | 0.496 |  | (3)(4) | 77.40 | 0.845 | 81.88 | 72.93 | 0.550 |
|  | (1)(2)(3) | 76.74 | 0.844 | 76.85 | 76.63 | 0.535 |  | (1)(2)(3) | 78.20 | 0.851 | 82.70 | 73.71 | 0.566 |
|  | (1)(2)(4) | 76.18 | 0.839 | 75.96 | 76.40 | 0.524 |  | (1)(2)(4) | 77.87 | 0.860 | 81.35 | 74.38 | 0.559 |
|  | (1)(3)(4) | 77.49 | 0.864 | 73.76 | 81.22 | 0.551 |  | (1)(3)(4) | 78.28 | 0.857 | 83.03 | 73.53 | 0.568 |
|  | (2)(3)(4) | 75.11 | 0.821 | 72.27 | 77.95 | 0.503 |  | (2)(3)(4) | 78.28 | 0.857 | 82.31 | 74.24 | 0.567 |
|  | (1)(2)(3)(4) | 77.82 | 0.851 | 74.77 | 80.86 | 0.557 |  | (1)(2)(3)(4) | 79.84 | 0.860 | 84.91 | 74.77 | 0.600 |
| RF | (1)(2) | 80.67 | 0.875 | 87.87 | 73.48 | 0.620 | SVM | (1)(2) | 78.31 | 0.865 | 82.92 | 73.71 | 0.569 |
|  | (1)(3) | 81.26 | 0.880 | 86.68 | 75.85 | 0.629 |  | (1)(3) | 78.44 | 0.864 | 80.81 | 76.07 | 0.569 |
|  | (1)(4) | 80.54 | 0.870 | 86.20 | 74.89 | 0.615 |  | (1)(4) | 77.04 | 0.850 | 80.77 | 73.30 | 0.542 |
|  | (2)(3) | 81.05 | 0.863 | 87.89 | 74.22 | 0.627 |  | (2)(3) | 78.48 | 0.860 | 80.49 | 76.46 | 0.570 |
|  | (2)(4) | 79.01 | 0.870 | 83.30 | 74.73 | 0.582 |  | (2)(4) | 79.12 | 0.860 | 81.76 | 76.48 | 0.583 |
|  | (3)(4) | 79.91 | 0.860 | 84.28 | 75.55 | 0.601 |  | (3)(4) | 77.51 | 0.833 | 75.98 | 79.04 | 0.550 |
|  | (1)(2)(3) | 81.12 | **0.883** | 88.54 | 73.71 | 0.629 |  | (1)(2)(3) | 79.78 | 0.869 | 83.15 | 76.40 | 0.597 |
|  | (1)(2)(4) | 80.11 | 0.876 | 86.74 | 73.48 | 0.608 |  | (1)(2)(4) | 78.76 | 0.866 | 83.82 | 73.71 | 0.578 |
|  | (1)(3)(4) | **83.03** | 0.882 | **88.69** | 77.38 | **0.665** |  | (1)(3)(4) | 78.51 | 0.868 | 81.45 | 75.57 | 0.571 |
|  | (2)(3)(4) | 80.79 | 0.874 | 86.24 | 75.33 | 0.619 |  | (2)(3)(4) | 78.17 | 0.858 | 78.17 | 78.17 | 0.563 |
|  | (1)(2)(3)(4) | 80.97 | 0.878 | 86.71 | 75.23 | 0.623 |  | (1)(2)(3)(4) | 79.28 | 0.870 | 83.11 | 75.45 | 0.587 |

(1): GPSD (2): ASDC (3): GAAPC (4): PAAC

1. **Additional Figures**


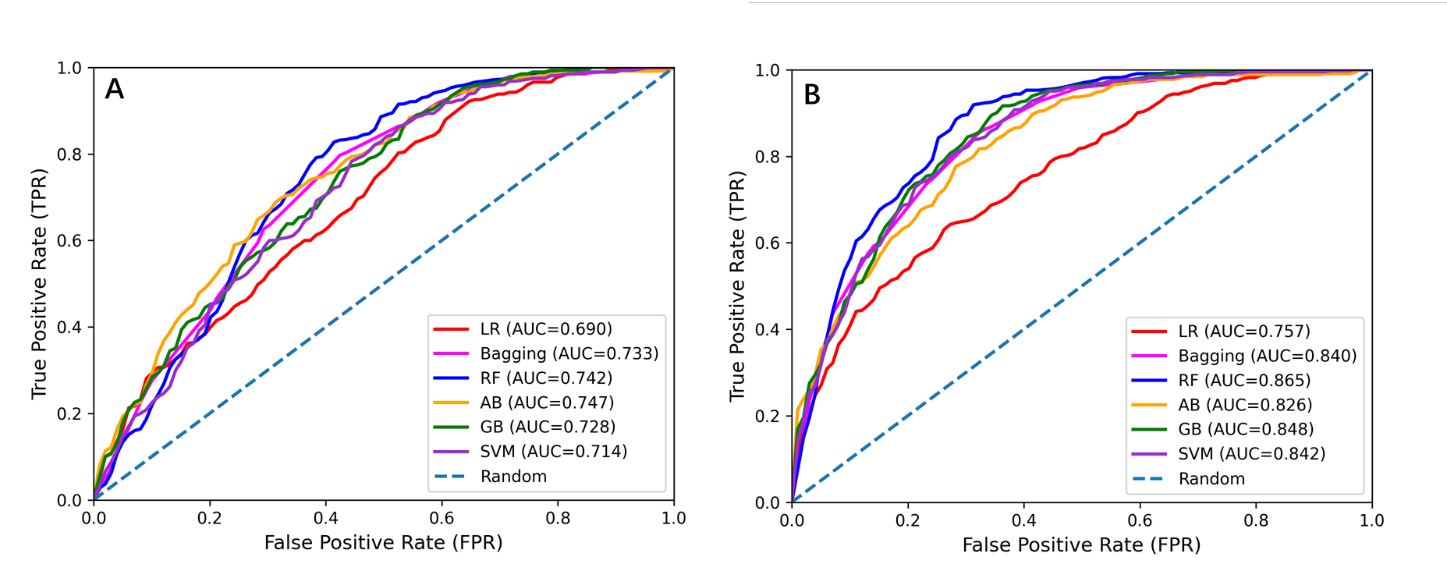


**Figure S1** 10-fold CV ROC curves for GPSD descriptor on six classifiers: (**A**) without SMOTE-Tomek; (**B**) with SMOTE-Tomek.


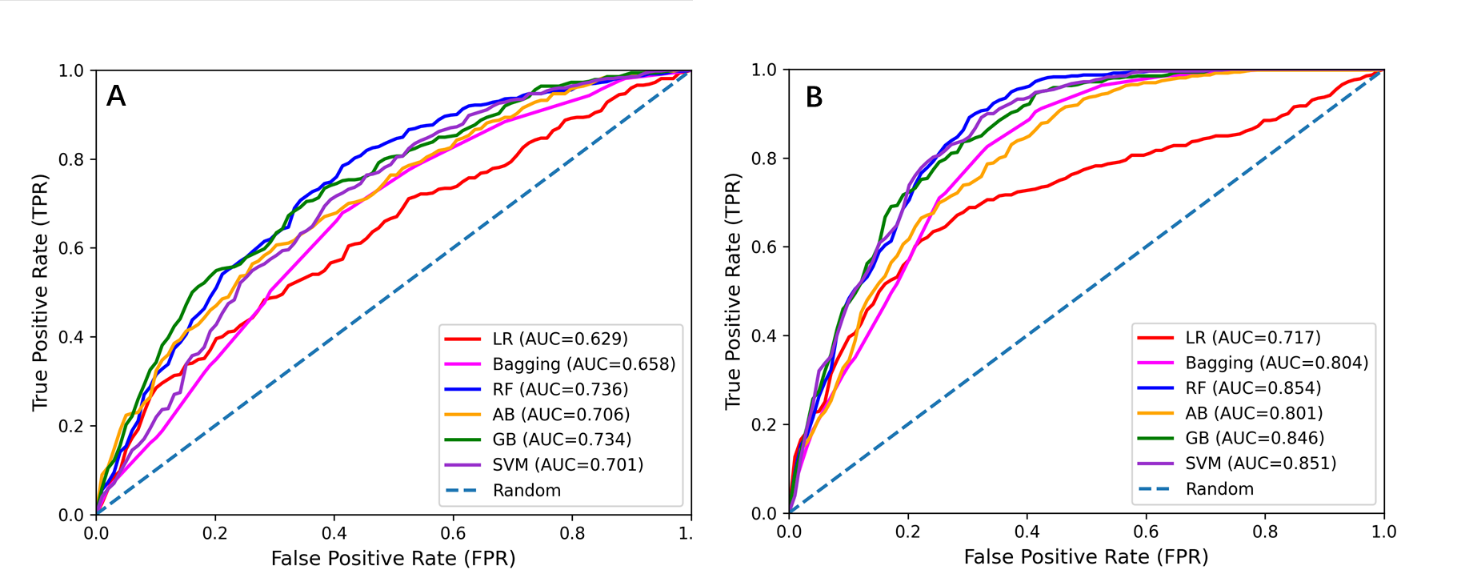


**Figure S2** 10-fold CV ROC curves for ASDC descriptor on six classifiers: (**A**) without SMOTE-Tomek; (**B**) with SMOTE-Tomek.


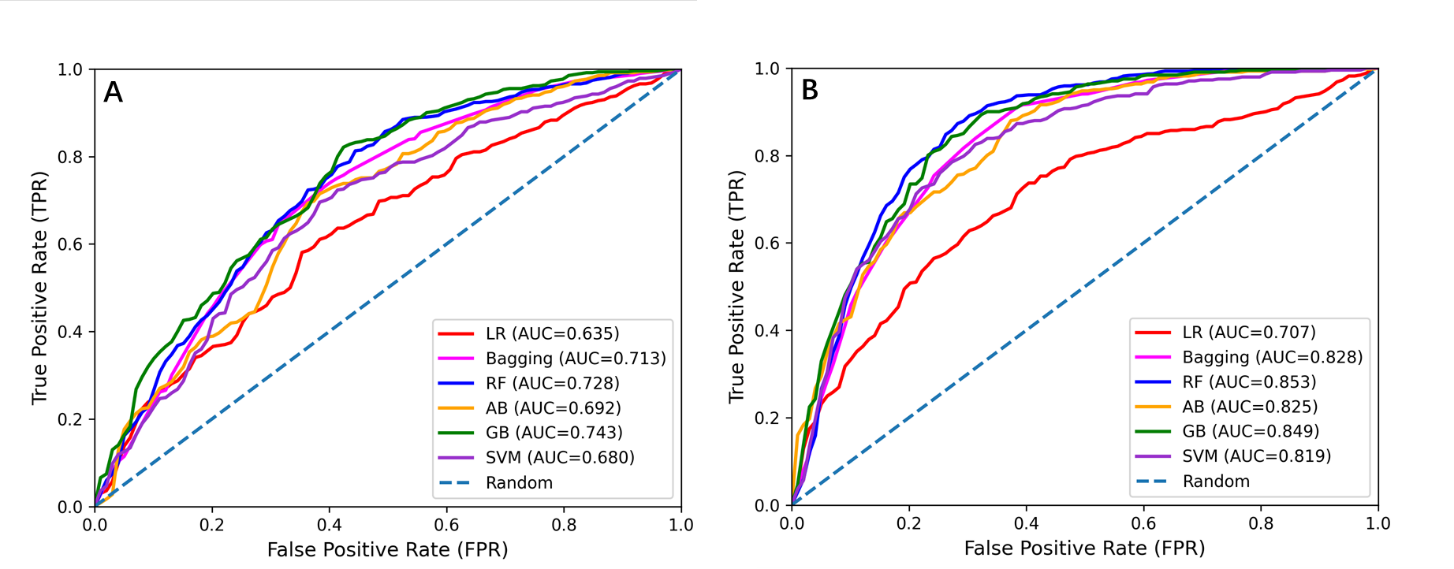


**Figure S3** 10-fold CV ROC curves for GAAPC descriptor on six classifiers: (**A**) without SMOTE-Tomek; (**B**) with SMOTE-Tomek.


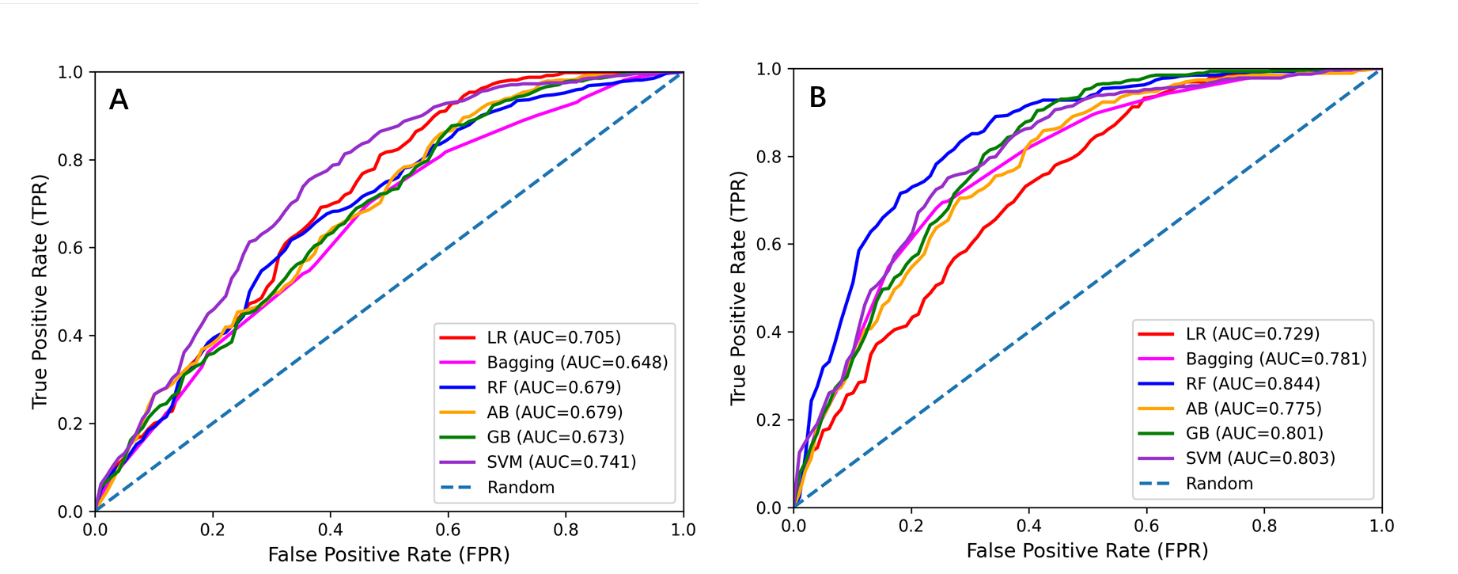


**Figure S4** 10-fold CV ROC curves for PAAC descriptor on six classifiers: (**A**) without SMOTE-Tomek; (**B**) with SMOTE-Tomek.
